# Landscape requirements of a primate population in a human-dominated environment

**DOI:** 10.1186/1742-9994-9-1

**Published:** 2012-01-23

**Authors:** Tali S Hoffman, M Justin O'Riain

**Affiliations:** 1 Zoology Department, University of Cape Town, Private Bag X3, Rondebosch 7701, South Africa

**Keywords:** habitat selection, human-wildlife conflict, non-human primates, spatial ecology, wildlife conservation, wildlife management.

## Abstract

**Introduction:**

As urban and rural land development become widespread features of the global landscape so an understanding of the landscape requirements of displaced and isolated wildlife species becomes increasingly important for conservation planning. In the Cape Peninsula, South Africa, rapid human population growth, and the associated urban and rural land transformation, threatens the sustainability of the local chacma baboon population. Here we analyse spatial data collected from nine of the 12 extant troops to determine their population-level landscape requirements. We use hurdle models to ascertain the key landscape features influencing baboon occurrence and abundance patterns on two hierarchical spatial scales.

**Results:**

Both spatial scales produced similar results that were ecologically reliable and interpretable. The models indicated that baboons were more likely to occur, and be more abundant, at low altitudes, on steep slopes and in human-modified habitats. The combination of these landscape variables provides baboons with access to the best quality natural and anthropogenic food sources in close proximity to one another and suitable sleeping sites. Surface water did not emerge as an influential landscape feature presumably as the area is not water stressed.

**Conclusions:**

The model results indicate that land development in the Cape Peninsula has pushed baboons into increasingly marginal natural habitat while simultaneously providing them with predictable and easily accessible food sources in human-modified habitats. The resultant spatial competition between humans and baboons explains the high levels of human-baboon conflict and further erosion of the remaining land fragments is predicted to exacerbate competition. This study demonstrates how the quantification of animal landscape requirements can provide a mechanism for identifying priority conservation areas at the human-wildlife interface.

## Introduction

The primary goal of ecologists is to understand the ecological factors that determine species distribution and abundance patterns [1]. Furthermore, as urban expansion and rural land development become more widespread on the global landscape [2], so the understanding of the spatial requirements of species becomes increasingly important for conservation planning and management [3].

### Primate conservation

Habitat domination by humans [4], and the concomitant compression, fragmentation and conversion of primate habitats [5], are the driving forces behind human-primate conflict and one of the greatest threats to primate survival [6]. The use of space has thus become a central theme in primate studies [7], with conservationists relying on patterns of habitat use and minimum resource requirements for the effective conservation and management of various primate populations [7]. This is particularly true for those inhabiting small, isolated and fragmented habitats [8].

Within primates, baboons (genus *Papio*) are among the genera exhibiting the greatest degree of spatial overlap with humans [9]. This success is attributed to their agility, dexterity, high levels of sociality and co-operation, combined with dietary and behavioural flexibility [10]. Like the *Macaca *and *Cercopithecus *genera [9,11], baboons can survive, and even appear to thrive, in human-modified habitats [12]. However, as human populations expand and more land is developed, so the benefits afforded to baboons by habitat alteration are likely to be exceeded by the deleterious consequences of competition for space [6].

The chacma baboon population in the Cape Peninsula, South Africa provides one of the best examples of primate commensalism with humans. The earliest records of baboon and human co-existence in the Cape Peninsula date back to the 15^th ^century with the arrival of Dutch settlers in South Africa [13]. Over the last century the human population has grown rapidly [14], and all Cape Peninsula troops now have contact with humans, albeit to different degrees, in both residential and tourist-frequented areas. Furthermore, nearly half of the Cape Peninsula landscape has been transformed by a combination of urbanisation, farming and invasions by self-sown alien vegetation. Despite reductions in available land, geographical isolation caused by urban sprawl, historical extirpation of troops [15] and current high levels of human-induced injury and mortality [16], the local baboon population has persisted and has shown a steady increase in size over the last decade from 365 [1998; [17]] to 475 [2011; Beamish E.K., University of Cape Town, unpublished information].

Of concern to the sustainability of the Cape Peninsula baboon population are the continued expansion of the human population and the increasing spatial extent of the city of Cape Town, both of which have doubled over the last 30 years [14]. In addition to exacerbating already high levels of human-baboon conflict, further encroachment of humans into natural areas of the Cape Peninsula may compromise baboon conservation in several ways. First, the spatial concentration of the baboon population may make it susceptible to intra-specific infectious diseases [18]. Second, increasing overlap of baboons and humans could heighten the probability of bidirectional interspecies disease transmission [19,20]. Third, the survivability and ecological role of future generations of baboons could be compromised if young baboons that grow up in troops heavily reliant on human food sources do not learn the necessary skills for finding and processing indigenous food [21]. Finally, as contact between humans and baboons increases so baboons may become increasingly aggressive towards humans, as observed for vervet monkeys [*Cercopithecus aethiops*: [11]] and macaques [*Macaca thibetana*: [22]], and in extreme cases may be euthanized to protect public health and safety.

**Figure 1:**
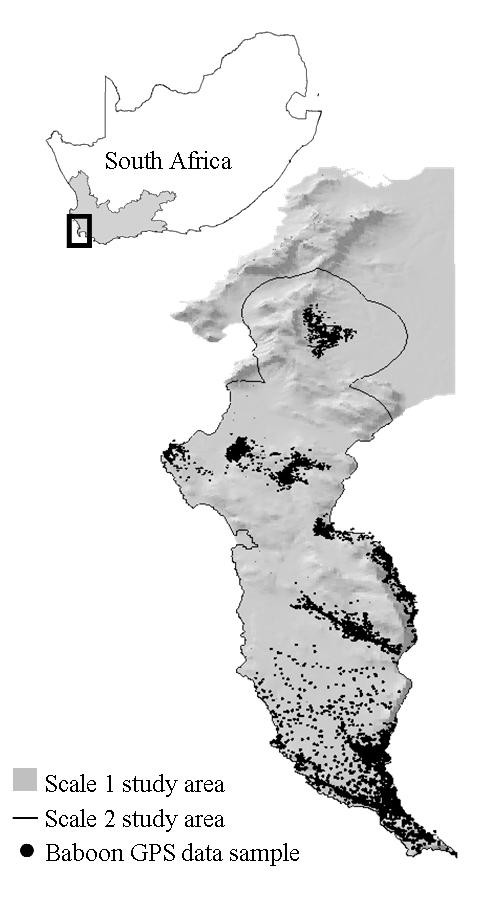
**Geographical location of the model study areas**. A map of South Africa (inset) with the black block indicating the Cape Peninsula in the Western Cape Province (shaded grey). The larger map shows the Scale 1 (entire grey area) and Scale 2 (within the black outline) study areas used in the hurdle models, and includes the sample of baboons GPS datapoints (*n = *9000) used in the models.

The first step in devising a conservation plan for a sustainable population is to encompass the landscape requirements of baboons in the spatial planning processes of land development in the Cape Peninsula. Current knowledge of baboon landscape requirements is speculative and anecdotal and here we use spatial data from nine troops to determine population-level patterns of landscape selection. We use hurdle models to ascertain the key landscape features influencing baboon occurrence and abundance patterns on two hierarchical spatial scales (Figure 1) [23]. We incorporate the foundations of primate ecological theory into the modelling process by selecting landscape variables (namely: habitat, altitude, slope and water) that provide baboons access to the three resources critical to their survival: food [e.g., [24]], sleeping sites [e.g., [25,26]] and water [e.g., [27]]. To enhance the ecological interpretability of the model results we conduct two post-hoc investigations to assess the spatial relationships between altitude and slope, and altitude and vegetation biomass. Finally, we investigate the implications of baboon landscape requirements for the current and future conservation of this geographically isolated and locally fragmented population by using the model results to calculate the absolute and cumulative areas of natural habitat remaining in the Cape Peninsula relative to baboon occurrence and abundance patterns.

## Results

### Occurrence

The models developed for the two spatial scales (Scale 1 and Scale 2) detected the same relationships between baboon occurrence and the ecological predictors (Tables 1 and 2). Habitat had the greatest influence on baboon occurrence, followed by distance to water, slope and then altitude. Among the topographic variables, the probability of baboon occurrence increased significantly with increasing distance to water, increasing slope and decreasing altitude. Within the habitat predictor variable, and relative to natural habitat (reference category), the probability of baboon occurrence increased significantly in agricultural habitat and invasive alien vegetation and decreased in urban habitat.

**Table 1:** Scale 1 model results

	Occurrence model coefficients				Abundance model coefficients			
Predictors	Estimate	1SE	z	p(> |z|)	Estimate	1SE	z	p(> |z|)
*Natural (intercept)*	-2.484	0.058	-42.797	< 0.001*	-8.137	18.380	-0.443	0.658
*Agriculture*	1.814	0.080	22.531	< 0.001*	-0.075	0.146	-0.512	0.609
*Invasive alien*	1.279	0.124	10.343	< 0.001*	0.962	0.223	4.320	< 0.001*
*Urban*	-1.741	0.085	-20.469	< 0.001*	0.581	0.166	3.490	< 0.001*
								
Altitude	-0.004	0.000	-14.582	< 0.001*	-0.005	0.001	-5.949	< 0.001*
Slope	0.021	0.003	6.733	< 0.001*	0.052	0.007	7.745	< 0.001*
Water	0.500	0.014	36.448	< 0.001*	-0.037	0.023	-1.619	0.105
								
Log (theta)					-10.450	18.380	-0.569	0.570

Results of the Scale 1 occurrence and abundance models including the coefficient estimates, standard errors (1 SE), z-statistics and p values for each predictor. Habitat categories are italicised and significant values (p < 0.05) are marked with *.

**Table 2:** Scale 2 model results

	Occurrence model coefficients				Abundance model coefficients			
Predictors	Estimate	1SE	z	p(> |z|)	Estimate	1SE	z	p (> |z|)
*Natural (intercept)*	-2.329	0.060	-38.994	< 0.001*	-8.766	25.170	-0.348	0.728
*Agriculture*	1.626	0.083	19.633	< 0.001*	-0.074	0.146	-0.511	0.609
*Invasive alien*	1.063	0.125	8.522	< 0.001*	0.962	0.223	4.319	< 0.001*
*Urban*	-0.717	0.087	-8.214	< 0.001*	0.581	0.166	3.489	< 0.001*
								
Altitude	-0.004	0.000	-13.748	< 0.001*	-0.005	0.001	-5.953	< 0.001*
Slope	0.027	0.003	8.282	< 0.001*	0.052	0.007	7.749	< 0.001*
Water	0.489	0.016	31.4	< 0.001*	-0.037	0.023	-1.615	0.106
								
Log(theta)					-11.080	25.170	-0.44	0.660

Results of the Scale 2 occurrence and abundance models including the coefficient estimates, standard errors (1 SE), z-statistics and p values for each predictor. Habitat categories are italicised and significant values (p < 0.05) are marked with *.

**Figure 2:**
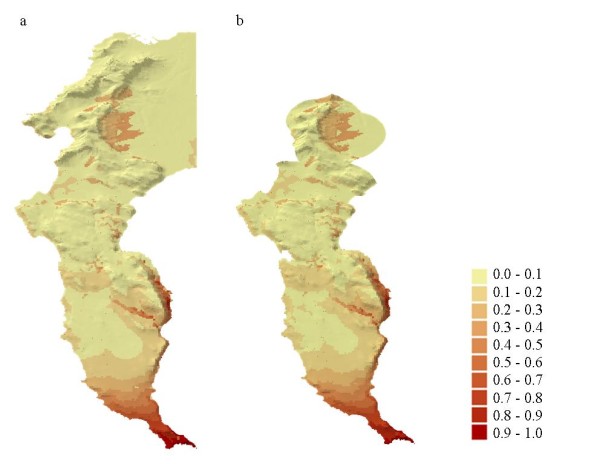
**Mapped predictions of baboon occurrence in the Cape Peninsula**. Predicted probabilities of baboon occurrence derived from the Scale 1 model (a) and the Scale 2 model (b).

The greatest difference between the two models was the magnitude of the coefficient estimate for urban habitat. Urban habitat had a stronger negative effect on baboon occurrence at Scale 1 compared to Scale 2. This difference is minimally evident in the occurrence probability maps for the two spatial scales (Figure 2).

### Abundance

The Scale 1 and Scale 2 models found the same relationships between baboon abundance and the predictor variables (Tables 1 and 2). Habitat had the greatest influence on baboon abundance, followed by slope and then altitude. Among the topographic variables, the predicted abundance of baboons increased significantly with increasing slope and decreasing altitude. Relative to habitat, and compared to natural habitat, the predicted baboon abundance increased significantly in invasive alien vegetation and urban habitat. Distance to water and agricultural habitat had no significant influence on baboon abundance.

**Figure 3:**
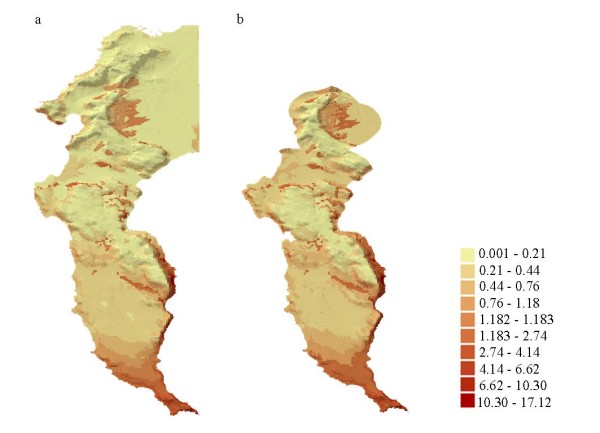
**Mapped predictions of baboon abundance in the Cape Peninsula**. Predicted values of baboon abundance derived from the Scale 1 model (a) and the Scale 2 model (b).

The similarity of the results produced by the Scale 1 and Scale 2 models - in magnitude and significance - meant that the same ecological conclusions could be drawn from both models. However, small differences in model performance abilities (see *Methods*), model dispersion parameters (theta; the shape parameter of the negative binomial distribution) and model statistics (Tables 1 and 2) meant that the models differed in their predictions of abundance relative to the predictors (see *Methods*). Consequently, the maps of predicted abundance differ noticeably, with the Scale 2 model (Figure 3b) predicting a more generous abundance of baboons across the Cape Peninsula landscape (abundance values > 0.21) than the Scale 1 model (Figure 3a).

### Post-hoc investigation results

**Figure 4:**
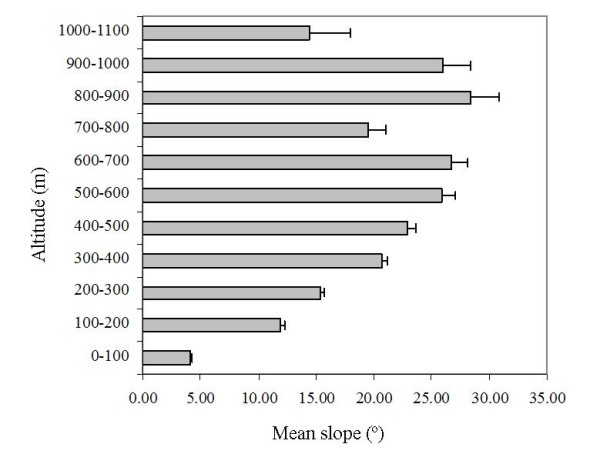
**Slope and altitude characteristics in the Cape Peninsula**. The mean (± 1SE) slope of all altitudinal belts in the Cape Peninsula.

To better understand these model results in the context of the Cape Peninsula landscape we conducted transect surveys to investigate the spatial relationships between altitude and slope, and altitude and vegetation biomass (see *Methods*). Across the three surveyed transects the largest plants were found in the lowest altitudinal belts (Table 3). Low trees (< 10 m) and large shrubs (> 2 m) dominated the lower elevations, with plant height decreasing at altitudes ≥ 400 m. Plant cover remained consistent at all altitudes despite the decrease in plant height. Slope was lowest in the lowest altitudinal belt (Figure 4) and apart from a decrease at the 700-800 m belt, slope increased steadily to 900 m, decreasing thereafter.

**Table 3:** Altitudinal vegetation characteristics

	Transect 1		Transect 2		Transect 3	
Altitude	Height	Cover	Height	Cover	Height	Cover
500-600 m	Shrubs 1-2 m	75-100%	Shrubs 1-2 m	75-100%	Shrubs 1-2 m	75-100%
400-500 m	Shrubs 1-2 m	75-100%	Shrubs > 2 m	75-100%	Shrubs > 2 m	75-100%
300-400 m	Shrubs > 2 m	75-100%	Shrubs > 2 m	75-100%	Low trees < 10 m	75-100%
200-300 m	Shrubs > 2 m	75-100%	Low trees < 10 m	75-100%	Low trees < 10 m	75-100%
100-200 m	Shrubs > 2 m	75-100%	Low trees < 10 m	75-100%	Low trees < 10 m	75-100%
0-100 m	Shrubs > 2 m	75-100%	Low trees < 10 m	75-100%	Shrubs 1-2 m	75-100%

Vegetation height and cover (following [76]) of the altitudinal vegetation transects. Data are sorted from highest to lowest altitudes.

**Figure 5:**
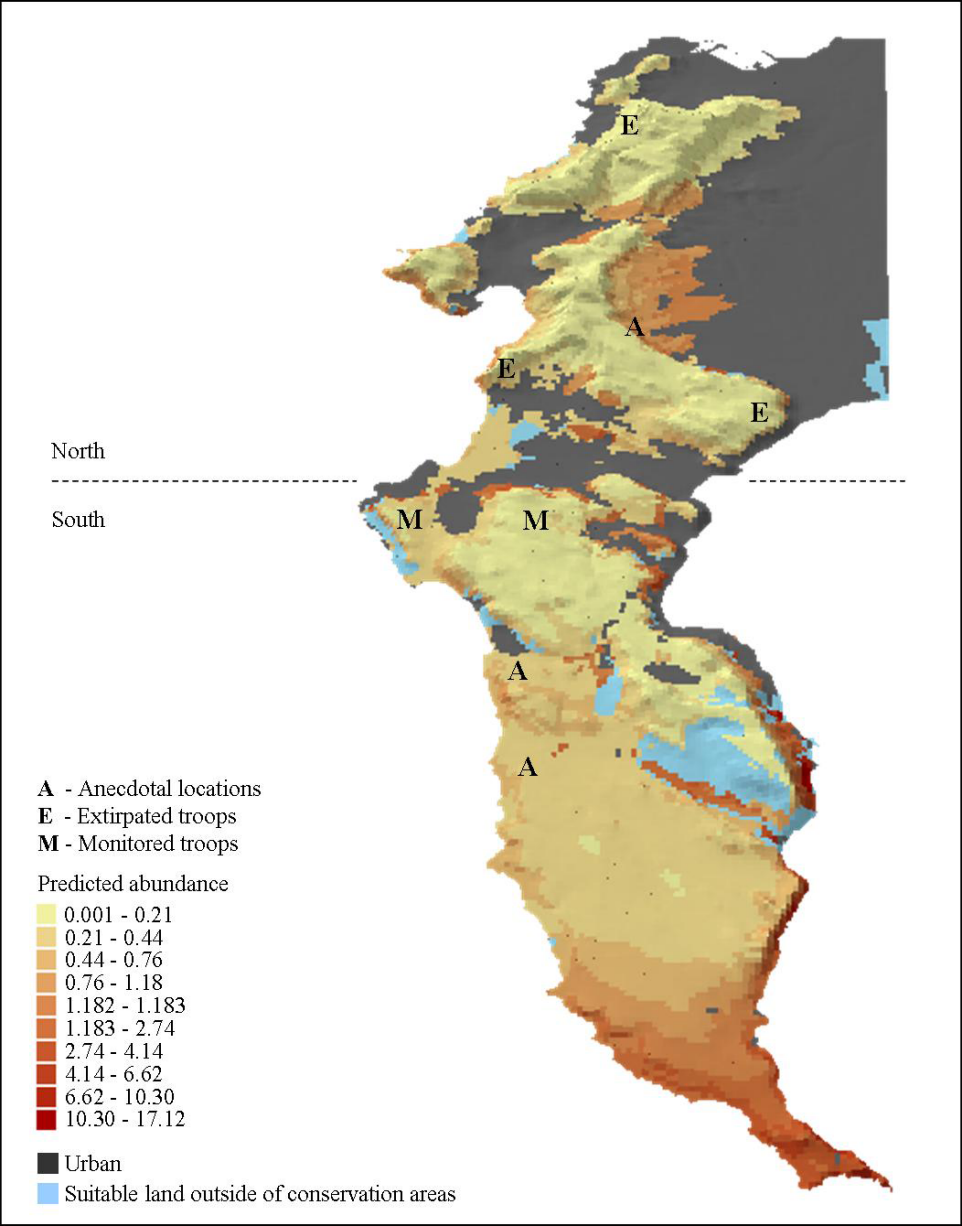
**Predicted baboon abundance, urban habitat and ecologically suitable land excluded from conservation areas**. The predicted abundance values from the Scale 1 model overlaid with the extent of urban habitat in the Cape Peninsula, as well as the areas of land suitable for baboons (probability of occurrence > 0.5) that are not currently conserved within the Table Mountain National Park. Included on the map are the locations of troops not included in this study (T), troops extirpated prior to this study (E) and troops monitored during this study (M). A belt of urban habitat (dashed line), situated approximately half way down the length of the Cape Peninsula, serves to divide baboons into northern and southern sub-populations.

There is only a minimal amount of natural habitat remaining that satisfies the landscape requirements of baboons (Figure 5; Table 4). Most of the natural habitat is available in areas that have a low probability of baboon occurrence (p < 0.05) and low levels of predicted abundance (< 5 GPS data points per cell).

**Table 4:** Cumulative and total area of remaining natural habitat

Occurrence probability			Predicted abundance		
Probability	Undeveloped area (km^2^)	Cumulative area (km^2^)	Abundance	Undeveloped area (km^2^)	Cumulative area (km^2^)
0.9-1.0	0.6	0.6	15-20	0.0	0.0
0.8-0.9	1.8	2.3	10-15	0.0	0.0
0.7-0.8	1.5	3.8	5-10	1.1	1.1
0.6-0.7	1.8	5.6	1-5	20.2	21.3
0.5-0.6	2.9	8.5	0-1	242.9	242.9
0.4-0.5	4.5	13.0			
0.3-0.4	4.3	17.3			
0.2-0.3	7.8	25.1			
0.1-0.2	43.1	68.2			
0.0-0.1	196.1	264.2			

Remaining area of natural habitat at each level of occurrence probability and predicted abundance, and including cumulative totals. Data are sorted in decreasing order of probability, and decreasing values of abundance.

## Discussion

### Ecological reliability and interpretability of models

While the complexity of biological systems inhibits the ability of ecological models to reflect all reality, a model that suitably approximates the information contained in empirical data allows interesting inferences about ecology to be made [28]. On account of their generalist nature [10], baboons are likely to have occurred throughout the Cape Peninsula prior to urbanisation, but with abundance being higher in more favoured habitat. The models reflect this pattern with the evaluation results and output maps showing that, despite being poorly correlated, the models provided qualitative predictions of baboon ranging patterns that can be considered accurate for four reasons. First, both the Scale 1 and Scale 2 models accurately predicted the current distribution of all studied troops. Second, both models predicted a higher abundance of baboons on land that is currently being used by troops that were not included in this study (n = 3; Figure 5) as well as historically recorded locations of extirpated troops (n = 3). Third, in only small, non-contiguous patches did the models predict baboon occurrence or abundance patterns that are not supported by either historic records or current baboon distribution patterns. Finally, the model predictions deviated most notably for the two troops most actively herded within their home ranges by baboon monitors [29]. Here, both the Scale 1 and Scale 2 models predicted a lower probability of occurrence and a lower predicted abundance than we would expect given the troops ranging patterns [SK and DG troops: [30]]. This would suggest that the herding of baboons by monitors has impacted on their habitat use.

The low levels of positive spatial auto-correlation in the model residuals resulted from underlying landscape patterns, and may explain the average calibration of the models. Spatial correlation is almost always present in grid-datasets [31,32] but as it does not bias regression coefficients [33] it does not affect the ecological interpretability of the models.

### Key landscape features

It is possible, based on the inherent properties of the landscape, that the mountainous spine that runs the length of the Cape Peninsula has never provided sufficient food resources to support a large, spatially continuous baboon population. Baboons may have always been reliant on access to low land to obtain sufficient food, with high altitude areas acting as a demographic sink for the expanding population [e.g., [34]]. Thus, the rapid growth of the human population and extensive urbanisation over the last two centuries [14] has not only isolated the local baboons from all other populations but has also annexed most of the low lying and more productive foraging areas. That baboons have continued to persist in this environment despite these challenges is testament to their ability to modify their foraging behaviour and coexist with humans. This coexistence, however, has come at a severe cost with whole troops having been extirpated [15] and frequent cases of human-induced injury and mortality [16].

Both models indicated that baboons are more likely to occur, and be more abundant, at low altitudes, on steep slopes and in some human-modified habitats. These patterns are congruent with predictions based on baboon ecology (see below) as the combination of these variables provides baboons with access to food and sleeping sites - two resources critical to their survival.

#### Food

Optimal foraging strategies for primates simultaneously maximise nutrient gain [35] and the efficient use of available time [36]. Accordingly, patterns of primate distribution and abundance across the landscape can be explained primarily by the distribution of the most lucrative foraging sites [37]. Baboon occurrence and abundance in the Cape Peninsula converged with the areas of the landscape that have the most profitable food sources, namely lower altitudes (see Table 3) and human-modified habitats.

##### Altitude and food

In the Cape Peninsula the benefits of foraging at low altitudes are threefold for baboons. Firstly, not only do the lower altitudes appear to contain larger plants than the rocky mountaintops - as revealed by landscape surveys - but the productivity of vegetation also decreases as altitude increases [A.R. Rebelo, South African National Biodiversity Institute, unpublished information]. Secondly, baboons gain access to high protein food resources along the coastline by consuming a variety of marine intertidal organisms. Not all local troops have access to the latter food source but troops that spend the majority of their time in the protein-poor indigenous vegetation [38] routinely include marine-food sources in their diet [Lewis M.C., University of Cape Town, unpublished information]. Thirdly, most human-modified habitat occurs at lower altitudes and offers highly concentrated and predictable food resources.

##### Human-modified habitats and food

Anthropogenic habitat alteration can dramatically affect the quality, availability and distribution of food resources and the addition of anthropogenic food sources into primate diets can have a positive effect on both their abundance and fecundity [39]. In addition to the food sources available in urban (e.g., fruit trees in gardens, garbage in refuse bins, food items in houses) and agricultural habitats (e.g., grapes in vineyards, pine nuts in *Pinus *plantations, ostrich feed in livestock farms), humans in the Cape Peninsula have introduced many species of invasive alien plants [e.g., *Pinus, Acacia *and *Eucalyptus *spp., [40]] which have both higher seed production and standing biomass than indigenous vegetation [41]. Human-modified habitats thus offer abundant, accessible and calorie-rich food sources that baboons favour over the low quality forage of local indigenous vegetation [29,42]. That the models detected, on average, that baboons preferred human-modified habitats to natural habitat is thus unsurprising. There were, however, some interesting exceptions to this trend.

##### Urban habitat

Baboons were less likely to occur in urban than in natural habitat despite the abundance of high quality food sources available in both houses and gardens. This is almost certainly a consequence of conflict with humans, with baboons suffering from harassment, injury and mortality when foraging in urban habitat [16]. However, in time baboons are able to adapt to profitable foraging conditions by improving their raiding success while simultaneously minimising the costs associated with foraging in high risk habitats [5]. Indeed baboons in the Cape Peninsula manage to mitigate against human threats while maximizing nutrient gain by spending minimal time raiding in urban habitat, acquiring human food quickly and returning thereafter to the relative safety of other habitats [29]. This raiding strategy would explain the low probability of occurrence predicted for urban habitat.

However, an additional explanation for these results may lie in the statistical procedure of the modelling process. In both the Scale 1 and Scale 2 models the proportion of urban habitat used was much lower than what was available. When the amount of available urban habitat was decreased relative to the habitat use values (Scale 2 model) the negative effect of urban habitat on baboon occurrence was reduced. This suggests a sensitivity of the occurrence models to large discrepancies between use and availability values. With these discrepancies controlled, as they were in the abundance models, urban habitat was found to be favourable to natural habitat at both spatial scales.

##### Agricultural habitat

A similar pattern was found for agricultural habitat where the occurrence models determined that baboons preferred this habitat to natural habitat, but the abundance models found the preferences for the habitats to be similar. There are two possible - perhaps interacting - explanations for this pattern. First, some crops (e.g., vineyards) have a distinctly seasonal growth cycle and are consequently not used consistently by baboons on an annual basis [42]. Second, agricultural habitats are the source of income for farmers who, across sub-Saharan Africa consider baboons to be pests, capable of more crop damage than any other primates [43,44] or indeed any other wildlife species [45]. Of the types of agricultural habitat in the Cape Peninsula, baboons are tolerated in pine and eucalyptus plantations, but vineyard owners actively chase baboons when the vines are in fruit [42], and livestock farmers routinely chase baboons off their property throughout the year [29]. Seasonal differences in crop use and farm policing would not affect the presence-absence analyses of the occurrence models, but would reduce the significance of the overall patterns of baboon abundance in agricultural habitat.

##### Invasive alien vegetation

Both the occurrence and abundance models revealed that baboons found invasive alien vegetation to be significantly preferential to natural habitat. This result is unsurprising given the 3-10 fold increase in above-ground biomass associated with invasive alien vegetation [46] and the resultant preference for foraging in this habitat [29]. Furthermore, unlike urban habitat, baboons are able to exploit invasive alien vegetation without the cost of human harassment.

#### Sleeping-sites

While food resources play a crucial role in determining primate spatial distributions, their availability to the animals is constrained by their proximity to other critical resources. In affording baboons safety from predators [47] and providing them with suitable vantage points for area surveillance [48], sleeping-sites fundamentally affect baboon ranging patterns and dictate the intensity to which they use the landscape [26]. Baboons use a variety of sleeping-sites including cliffs, trees, and caves [27]. In the Cape Peninsula baboons sleep primarily in trees and on cliffs [[29,30]].

The baboons' use of trees as sleeping-sites provides an additional explanation for the preference shown for invasive alien vegetation and agricultural habitat over natural habitat. Natural habitat in the Cape Peninsula is characterised by shrublands, grasslands and low trees [38]. By contrast both self-sown invasive alien vegetation and cultivated plantation trees are suitably sized for baboon sleeping-site requirements [29,42]. Furthermore, plantation trees have also been cultivated alongside vineyards and urban areas and, because of the high levels of disturbance at the urban/natural habitat interface and the significant source of alien propagules presented by suburban gardens [40], self-sown alien plants tend to invade and establish in close proximity to urban habitat. Thus the spatial distribution of tall trees in the Cape Peninsula provides baboons with suitable sleeping-sites in close proximity to favoured foraging areas.

The importance of cliff sleeping-sites was also detected by the models. Both models indicated that baboons are more likely to occur, and be more abundant, on steep slopes - the inaccessibility of which provides them with a safe night-time refuge. The coincident preference for steep slopes and low altitudes once again represents the importance of proximity of sleeping-sites to favoured foraging areas. In the Cape Peninsula, slope steadily increases from the 100 m contour line (Figure 4). Because urban development is constrained by the exposure and inaccessibility of high altitudes and steep slopes [49] the spatial extent of urban habitat is restricted to the flat land below the 80 m contour line. Consequently steep cliffs, suitable as baboon sleeping-sites, occur directly above favourable urban habitat in many areas.

#### Water

Given that water is a critical resource for baboons it is surprising that there was a significantly higher probability of baboons occurring far from permanent surface water sources than near to them. An explanation for this pattern emerges when results are considered in context with the hydrological attributes of the Cape Peninsula. With permanently flowing surface waters, the presence of freshwater wetlands and vegetation prone to seasonal water-logging [38], the Cape Peninsula is not a water-stressed environment. These factors explained the relative lack of importance of water in determining the ranging patterns of one of the local troops [TK troop: [42]]. For this troop the high water content of vegetation explained the lack of 'drinking sessions' [*sensu *[27]] where many baboons converge at a waterhole and drink simultaneously. These explanations seem equally applicable to all troops in the Cape Peninsula, especially given the results of the abundance models that indicated no significant relationship between baboon land use and surface water. Thus, rather than revealing an interesting ecological phenomenon where animals avoid water, the model results should rather be interpreted as an indication that permanent surface water need not be considered as a key landscape feature for baboons in the Cape Peninsula during years of good rainfall. However, permanent water sources may indeed become a good predictor of baboon occurrence during years of drought or below par rainfall, particularly during the dry summer season.

## Conclusions

Currently, the most widely used method of baboon management is the employment of baboon monitors who attempt to reduce levels of human-baboon conflict by preventing troops from crossing the urban edge [29]. Because of the spatial attributes of urbanised areas, monitors must typically herd baboons away from favourable low lying land and up the mountain, into increasingly marginal habitat (Figure 5). That monitored troops still suffer extraordinary high levels of human-induced injury [16] is not attributable to atypical or errant baboon behaviour, but rather indicative of the intensity of the competition between baboons and humans for low lying land and the associated high quality natural and anthropogenic food resources. The inevitable persistence of this competition through time, will forever compromise the ability to effectively manage baboons at the interface of natural and urban habitats.

The implications of extensive land development for the future of baboon management and conservation are equally concerning. Currently, minimal amounts of the land considered to be ecologically suitable for baboons (probability of occurrence > 0.5) remain undeveloped. This is most pronounced in the northern half of the Cape Peninsula (Figure 5) where suitable natural habitat comprises non-contiguous islands made inaccessible to baboons by the sea of urbanisation surrounding them. The landscape of the southern half of the Cape Peninsula, where three-quarters of the current baboon population range, holds more promise with continuous stretches of ecologically suitable natural habitat still available. Of the natural habitat available in the southern Cape Peninsula, 87% is conserved as part of the Table Mountain National Park, with 13% potentially subject to urban or agricultural transformation. Transformation of this land will force baboons farther into the increasingly marginalised habitat of higher altitudes and will ultimately exacerbate levels of human-baboon conflict, with inevitable deleterious consequences for baboons. If attempts to conserve the Cape Peninsula baboon population are to be effective, then mitigating against the development of the remaining natural fragments of the landscape must be addressed urgently.

Conserving this undeveloped land will require the involvement of various landowners [50], including both private owners (62% of land) and national and provincial government (38%). The local government departments responsible for approving developments have already accepted the GPS data from this research and included then within new zoning schemes. While this will not automatically prevent further erosion of natural habitats, it will trigger baboon-specific Environmental Management Plans and initiate assessments of the possible impacts of such development on the affected troop(s), with input from interested and affected parties. However, because most undeveloped land is owned by private parties, it is vital that the responsibility of landscape conservation is not left entirely to environmental authorities. Rather, a great deal of effort should be placed on encouraging and or providing incentives for private landowners to participate in the conservation process. This can be achieved using a variety of methods including one or a mix of voluntary, property-based, price-based or regulatory mechanisms [51,52]. Ultimately, however, the effectiveness and sustainability of any landscape conservation strategy relies on the a combination of 'top-down rigour' and 'bottom-up participation' [53], where partnerships between management authorities, non-governmental organisations and private parties will ensure that land development plans are not only ecologically sustainable, but socially sustainable [54-56]. In line with this, a baboon liaison group was established in the Cape Peninsula in 2010 to serve as an intermediary between baboon management authorities, researchers and local communities. In providing a platform for communication, the establishment of this liaison group will improve the chances of the local baboon population being sustainable on the long-term.

This study highlights the complexities of wildlife management and conservation at the interface of natural and human-modified habitats. This is particularly true for wildlife whose landscape requirements are concurrent with those of humans, and whose ecological flexibility allows them to thrive in human-modified habitats - qualities which may increase their probability of experiencing conflict with humans. However, by enhancing our understanding of the fundamental drivers of human-wildlife conflict, the quantification of animal landscape requirements can support and validate wildlife conservation efforts. Furthermore, an understanding of animal spatial ecology can assist in discriminating between the relative importance of landscape quality and landscape quantity, and can provide a mechanism for identifying priority conservation areas at the human/wildlife interface.

## Methods

### Study site

Located at the south-western most point of the African continent (latitude 33°55'-34°21' S; longitude 18°25'-18°28 E), the Cape Peninsula (Figure 1) comprises a combination of natural and human-modified habitats bounded by the Atlantic Ocean. The topography is characterised by the Peninsula Mountain chain and has an altitudinal range of 0-1100 m. Lower elevations are predominantly urbanised, mid-elevations are used for agriculture (vineyards, plantations and livestock farming) and higher elevations are almost exclusively indigenous fynbos vegetation. Fynbos is a species-rich but nutrient poor, sclerophyllous shrubland that is a key component of the Cape Floristic Region [38]. More than half of the Cape Peninsula remains undeveloped and is conserved within the Table Mountain National Park. Chacma baboons are the only non-human primates present, and no natural baboon predators remain.

### Study population and data collection

At the time of data collection the Cape Peninsula baboon population comprised 12 troops ranging in size from 16-115 baboons [16]. Urban habitat and neighbouring troops served as the only barriers to troop movement and troops were able to range freely in approximately 250 km^2 ^of natural habitat. All nine study troops were accustomed to the presence of people and were habituated to close (≤ 10 m from baboons) observation at the start of the study. Four troops were managed by baboon monitors - people employed by the local management authorities to minimise human-baboon conflict by preventing troops from entering urban areas [29]. Troop home ranges show low levels of overlap (mean ± 1SE: 7.3 ± 4.9%) [30] and consequently troops seldom interact with each other. However, sub-adult and adult male baboons remain able to disperse to neighbouring troops in accordance with typical baboon behaviour.

Between 2006 and 2009 we collected one full year of spatial data for each of the study troops. Logistical and financial constraints prevented us from sampling all troops simultaneously, but there was no interannual variation in mean rainfall (*Kruskal-Wallis test*: H_(5, n = 2191) _= 6.6317, p = 0.250), mean maximum (ANOVA: F_5,2185 _= 0.8219, df = 2185, p = 0.0534) or mean minimum temperatures (ANOVA: F_5,2185 _= 1.5676, df = 2185, p = 0.166) across the study years. We recorded Global Positioning System (GPS) data points for each troop using (a) handheld devices (Garmin eTrex) operated by field researchers (n = 5 troops), (b) tracking collars (n = 3 troops) and (c) a combination of both methods (n = 1 troop). Field researchers recorded the GPS location of the centre point of the troop (visually estimated) at 20-minute intervals between sunrise and sunset for a mean of 109 days (± 28 days 1SE, range: 71-170 days, n = 6 troops) per troop. Tracking collars recorded the GPS point of a single troop member at 3-hourly intervals between sunrise and sunset for a mean of 302 days (± 54 days 1SE, range: 247-334 days, n = 3 troops) per troop. The terrain within the study area was easily traversable on foot and visibility of baboons within all habitat types was excellent. Only GPS data points that had an estimated level of accuracy of ≤ 10 m were included in our analyses. A total of 24,618 GPS data points was recorded for the population, with a mean of 2735 ± 768 GPS data points 1SE (range: 1668 - 5018, n = 9 troops) recorded per troop.

### Model study areas and datasets

**Figure 6:**
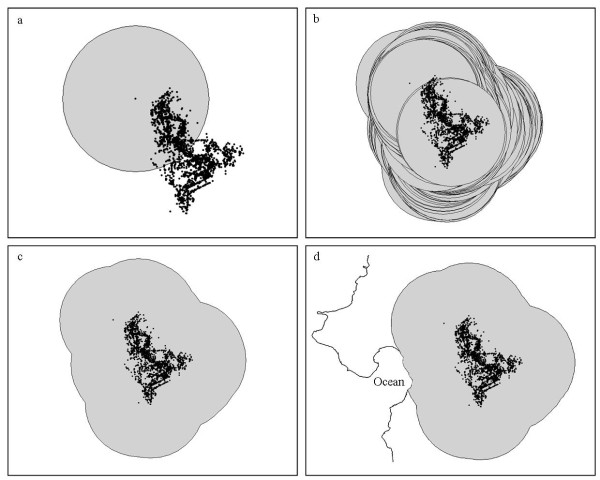
**Schematic of the steps followed to delineate the study area for the Scale 2 hurdle models**. (a) A circle of fixed diameter (buffer) was centred over a given GPS data point and (b) this process was repeated for all GPS data points collected throughout the study period. (c) The outermost extent of all buffers combined was used to produce an outline corresponding to the troops "accessible area". (d) If the accessible area extended beyond the Cape Peninsula landscape it was clipped to the coastline.

Confining studies to only one of the hierarchical scales at which landscape selection operates [57] may mask important aspects of landscape selection patterns [58]. Consequently we analysed landscape selection at two spatial scales which we selected based on their appropriateness for determining population-level management and conservation plans. The study area of the first spatial scale (Scale 1) covered 500.9 km^2 ^and spanned the full extent of the Cape Peninsula (Figure 1). To deduce patterns of landscape selection by baboons at the broadest level possible, this study area included land of all quality in the Cape Peninsula, irrespective of whether it was dominated by humans. The study area for the second spatial scale (Scale 2) covered 301.4 km^2 ^and included only land directly accessible to the troops that together comprise the population. We determined the Scale 2 study area following this same procedure for each troop. A circular zone (buffer) was placed around each GPS location (Figure 6). We defined the area contained within the outermost borders of the outlying buffers as the troops "accessible area". We based buffer radius lengths on the mean daily path length (mean ± 1SE: 4.0 ± 1.0 km) [30] traversed by each troop during their study period thereby representing a realistic measure of the area accessible to that troop within a day's journey from their home range (given that they typically return to known sleeping sites within their range). Accessible areas that extended beyond the extent of the Cape Peninsula landscape were clipped to the coastline. Spanning 301.4 km^2^, the Scale 2 study area included the combined extents of the accessible areas for all troops (Figure 1). While the Scale 2 model provided the more biologically meaningful scale - for it included patterns of baboon philopatry and spatial restrictions imposed by humans - the Scale 1 model was necessary for informing baboon management and conservation in the Cape Peninsula. Thus, as the models served different purposes, we have retained them both in this manuscript.

To produce tabular datasets for the models we assigned a matrix of grid cells to the Scale 1 and Scale 2 study areas. Each grid cell was 0.023 km^2 ^in area (150 m × 150 m) set to reduce the likelihood of any troop being spread through more than one cell simultaneously [59]. Consequently, the total area of each cell was sufficient to encompass the mean troop spread of the largest troop (mean ± 1SE: 0.021 ± 0.011 km^2^, n = 5 spreads). We merged GPS data from the baboon population to the Scale 1 and Scale 2 grids. The GPS data comprised 1000 GPS data points from each of the nine study troops, randomly selected to control for intertroop differences in sample sizes and sampling regimes, and the effects of group size and season on baboon ranging patterns. We pooled the GPS data together to generate a population-level dataset (Figure 1). For both study areas we determined a use value for each entered grid cell by counting the number of GPS data points within it. We assigned use-values of zero to non-entered cells.

For both model datasets we assessed over-dispersion by computing the ratio between the mean and variance of the data, where a variance much greater than the mean indicates over-dispersion [60]. We investigated zero inflation by calculating the percentage of zeroes present in each dataset. The variances of the count data were > 20 × larger than their respective means for both datasets (Table 5) indicating over-dispersion. Zero-inflation was present in both datasets, with zeroes accounting for 91.5% of the Scale 1 study area and 85.5% of the Scale 2 study area. On account of the sampling regime the source of this zero-inflation was not related to design, survey or observer error [false zeroes; [61]] but rather to the presence of structural (positive) zeroes resulting from cells being either suitable but not used, or unsuitable for use.

**Table 5:** Cell count details and predictor variable attributes of the model study areas

		**Scale 1 study area**		**Scale 2 study area**	
		
	**Area**	**500.9 km^2^**		**301.4 km^2^**	
Cell count details	Mean	0.39		0.66	
	Variance	8.27		13.9	
	% zeroes	91.5%		85.5%	
					
		Available	Used	Available	Used
Predictor attributes	Natural	52.7%	11.2%	65.3%	15.1%
	Agriculture	4.7%	27.5%	7.8%	27.8%
	Invasive alien	1.7%	29.9%	2.9%	30.0%
	Urban	41.0%	2.2%	23.9%	6.5%
					
		Mean ± SEM	Range	Mean ± SEM	Range
	Altitude	155.1 ± 2.4 m	0 - 1069.3 m	154.5 ± 2.5 m	0 - 911.81 m
	Slope	9.9.± 0.1°	0 - 61.8°	9.9.± 0.1°	0 - 57.4°
	Water	0.8 ± 0.02 km	0 - 10.2 km	1.1 ± 0.03 km	0 - 10.2 km

Area, mean (± 1 SE) and range (minimum-maximum) of topographic predictor variables, and percentage cover of habitat variables within the Scale 1 and Scale 2 study areas. Use values for the categorical predictors indicate the overall percentage of counts > 0 for each habitat type.

### Predictors

We analysed cell use as a function of the following predictor variables for both the Scale 1 and Scale 2 models: altitude (m), slope (°), the distance to permanent surface water sources (km) and habitat. We assigned explanatory variables (altitude, slope, distance to permanent surface water sources and habitat) to each cell in the Cape Peninsula grid matrix using the following methods. We calculated Altitude using a 30 m digital elevation model [DEM; Environmental Systems Research Institute. 1998. CSDGM FGDC Metadata DTD 3.0.0 19981217]. We used the same DEM to determine the slope of each cell, and calculated distances to permanent surface water sources using a shapefile [glcrveg; South African National Parks, unpublished information] that details drainage systems in the region. We used GIS maps containing landscape information specific to the Cape Peninsula (details below) to categorise habitat for each grid cell. In addition, we used observer records of location-specific habitats, and information gleaned from digitisation of the Cape Peninsula using Google Earth imagery to confirm and identify habitat in areas where the extent and or the detail of the GIS layers was insufficient or inaccurate in its descriptions. The percentage cover of each habitat within every cell was calculated using the Intersect Function of the Geoprocessing Wizard in ArcView 3.3. We converted the percentage values to categorical variables, and assigned habitat categories based on the dominant habitat (> 50% of cover) within each cell. We categorised habitat as natural, urban, agricultural and invasive alien vegetation. Natural habitat, categorised using a shapefile from [38], included indigenous vegetation, rocky shores and beaches. Urban habitat consisted of all urban areas delineated in the City of Cape Town's Generalised Zoning shapefile, cells with human-made structures such as buildings, gardens and grass patches adjacent to buildings, and cells dominated in cover by roads and sports fields. Agricultural habitat included plantations - delineated using a shapefile [lease_2006_06_28; South African National Parks, unpublished information] - and vineyards and an ostrich farm that were mapped digitally using Google Earth. We also used Google Earth to digitally map the range and extent of invasive alien vegetation (*Pinus, Acacia *and *Eucalyptus spp*.).

Ecological variables are frequently correlated with each other (multicollinear), and Pearson r values as low as 0.28 have the potential to bias analyses [62]. We used Pearson correlations to test for multicollinearity among predictor variables, with |r| > 0.28 set as the lower limit for multicollinearity [62]. Slope and altitude were positively correlated at |r| > 0.28 in both the Scale 1 and Scale 2 study areas (Table 6). Rather than minimising the biological importance of the models by excluding either variable [62], we regressed slope against altitude and subsequently replaced it with the residuals from the regression [63]. This procedure effectively removed the correlation between slope and altitude, with Pearson values of r < 0.001 for both the Scale 1 and Scale 2 models.

**Table 6:** Multicollinearity of predictor variables

Study area	Predictors	Altitude	Slope	Water
Scale 1	Altitude	-	0.62	-0.09
	Slope	0.62	-	-0.004*
	Water	-0.09	-0.004*	**-**
				
Scale 2	Altitude	-	0.55	-0.04
	Slope	0.55	-	0.11
	Water	-0.04	0.11	**-**

Pearson correlations indicating multicollinearity among continuous predictor variables in the Scale 1 and Scale 2 study areas. All correlation values are significant at p < 0.05 except for those marked with *.

The study areas differed in their overall composition of habitats but were similar in their topographic profiles (Table 5). We used both broad-scale and fine-scale habitat variables. The broad-scale variable (Broad Habitat) categorised habitat as being natural or human-modified. The fine-scale variable (Fine Habitat) included the broad-scale natural category and the human-modified sub-categories, namely urban habitat, agricultural habitat and invasive alien vegetation. To determine which of these habitat variables were most suitable for inclusion in the final models for each dataset, we evaluated each habitat variable in turn at the model building stage using the methods described under 'Model selection and evaluation' below. Once the final models were selected we evaluated each predictor in terms of its overall contribution to each respective model [64]. We used the habitat variable of 'natural' as the intercept category for both models.

### Statistical methods

#### Modelling algorithms and model fitting

For all datasets we used hurdle models [65] to analyse cell use as a function of the predictor variables. Potts and Elith [60] found that relative to four other regression models (Poisson, negative binomial, quasi-Poisson and the zero-inflated Poisson) the hurdle model had the greatest predictive performance when assessing the relationship between the abundance of an organism and its environment. Hurdle models are modified count models that separate data into two parts: one containing zero values and one containing positive counts [60]. As such, hurdles account for two ecological processes: the first is the process that causes an animal to be present at a site (occurrence) [61], and the second is the process that influences the numbers of animals found at a site, given that they occur there (abundance). Hurdle models model occurrence using binary (presence/absence) models with a binomial probability and model the abundance (positive counts) using zero-truncated count models [64]. Quasi-likelihood removes the effect of zero-inflation in the binary models and the effect of over-dispersion in the zero-truncated models [60].

Count data is typically modelled using the Poisson distribution, which assumes equality between means and variances [66]. However, actual count data are often over-dispersed (mean < variance) relative to the Poisson distribution [e.g., [67,61]]. In this instance it becomes more appropriate to use the negative binomial distribution, which allows for a quadratic relationship between the mean and the variance [e.g., [66,67]]. On account of the over-dispersion in both the Scale 1 and Scale 2 datasets (Table 5) we fitted the positive count models with negative binomial distributions.

We ran occurrence and abundance models for the Scale 1 and Scale 2 study areas. For the occurrence models we used a binomial distribution with logit link, and for the abundance models we used a negative binomial distribution with log link to ensure that the predicted values were always positive [8]. We conducted all statistical analyses using the R language and environment, an integrated software suite for statistical computing [68]. We fitted all models using the R package 'pscl' [69].

#### Model selection and evaluation

We used Akaike Information Criteria (AIC) to determine the best approximating hurdle model for the Scale 1 and Scale 2 study areas from a selection of candidate models (Table 7). We evaluated all candidate models in the same manner proposed by Potts and Elith [60] using correlation, calibration and error assessments. For correlations we determined both the Pearson correlation coefficient (r) and the Spearman rank correlation (R) for each model. Pearson's r indicates the relative agreement between observed and predicted values and Spearman's R indicates similarity in the ranks of the predicted and observed values [60]. Calibration, which describes the numerical accuracy of a model, relates the level of agreement between the models predicted values and the actual observations (goodness-of-fit) [70]. We assessed model calibration with a simple linear regression between the observed and predicted values [60]. A lack of agreement can be partitioned into bias (indicated by the intercept term; *b*) and spread (indicated by the slope of the line; *m*), where a perfectly calibrated model has *b *= 0 and *m *= 1 [71]. We used the average error (AVE_error_) and root mean squared error (RMSE) of the model residuals to assess discrepancies between predicted and observed values [60]. We generated graphs of the models to visually assess whether any structure was present in the relationships between Pearson residuals and the fitted counts and continuous predictor variables. We mapped the predicted values of baboon occurrence and abundance from the Scale 1 and Scale 2 models using ArcGIS 9.3 [Environmental Systems Research Institute, Redlands, California].

**Table 7:** AIC values for candidate models

Scale 1 candidate models	AIC	Scale 2 candidate models	AIC
FH and ALT and SL and WAT*	**18661.30**	FH and ALT and SL and WAT	**17722.50**
BH and ALT and SL and WAT	19932.58	BH and ALT and SL and WAT	18273.88
ALT and SL and WAT	19994.88	ALT and SL and WAT	18306.76
FH and ALT and SL	20115.03	WAT	18550.09
WAT	20186.56	FH and ALT and SL	18898.74
FH and ALT	20294.06	FH and ALT	19209.22
FH and SL	20815.91	BH and ALT and SL	19437.55
FH	20887.12	FH and SL	19473.10
BH and ALT and SL	21372.21	ALT and SL	19506.23
BH and ALT	21601.21	FH	19520.14
ALT and SL	21784.91	BH and ALT	19768.53
BH and SL	21885.26	ALT	19816.75
BH	21920.86	BH and SL	19852.92
ALT	22063.51	ALT:SL (interaction term)	19914.12
ALT:SL (interaction term)	22071.54	BH	19932.16

Akaike Information Criteria (AIC) values of all candidate models, sorted in ascending order for both spatial scales. Bold AIC values indicate the final models selected for each scale.

* ALT = Altitude; BH = Broad habitat; FH = Fine habitat; SL = Slope; WAT = Distance to water

#### Spatial autocorrelation

Spatial autocorrelation deals with a lack of independence of data points and measures the degree to which a variable is correlated to itself in space [72]. This phenomenon is pervasive in ecological datasets [73] and can stem from movement patterns of the study subject or underlying patterns of the landscape. Spatial autocorrelation can be problematic in analyses as it can lead to Type 1 statistical errors (false positives) and can result in inflated probabilities for predictor variables [74].

The random selection of GPS data points for inclusion in the model datasets accounted for any spatial autocorrelation attributable to animal movement patterns. During the modelling process, once the models are fitted to the data, predictor variables should account for any autocorrelation caused by landscape patterns. If this is not the case, then spatial autocorrelation should be evident in the model residuals [61]. We used GeoDa 0.9.5-i [GeoDa Center for Geospatial Analysis and Computation, Arizona] to test for spatial autocorrelation in the Scale 1 and Scale 2 model residuals using Monte Carlo simulation (999 permutations) of Moran's *I*. Moran's *I *ranges from -1.0 - 1.0, with non-zeroes indicating that the abundance values produced for spatially connected grid cells are either more similar (positive autocorrelation) or more different (negative autocorrelation) than would be expected given a random association among the cells [32]. We calculated Moran's *I *using a weight matrix defined by k-nearest neighbours, with the value for k defined by the number of cells within a 1 km radius from each cell (k = 224).

**Figure 7:**
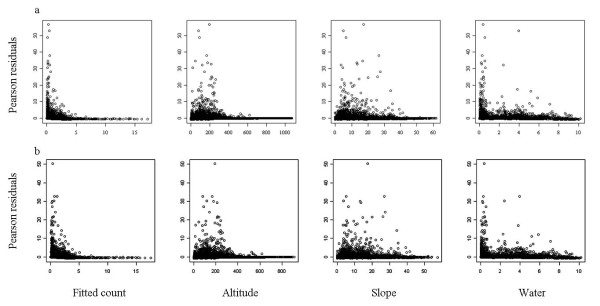
**Scale 1 and Scale 2 model diagnostics**. Diagnostics for the Scale 1 (a) and Scale 2 (b) hurdle models including plots of fitted counts and predictors against Pearson residuals.

We selected the candidate models that included the Fine Habitat variable as the final Scale 1 and Scale 2 models as they had lower AIC values than the models including the Broad Habitat variable (Table 8). Both models had consistently low levels of bias and were better calibrated than they were correlated. The amount of error around the predictions was low and when averaged across each study area was close to zero. However, even the small amounts of error might explain the low model correlation values which most likely resulted from error-related differences in observed and predicted values [60]. These errors, caused by variance in the models residuals, persist even under ideal sampling and analysis conditions [75]. Low but significant levels of spatial correlation were present in the residuals of the Scale 1 (Moran's *I *= 0.08, p < 0.01) and Scale 2 (Moran's *I *= 0.08, p < 0.01) models. Despite having the higher AIC of the two models, the Scale 1 model was the better performer in all evaluation tests barring the Spearman Rank correlations (Table 8). In both models, there was no structure present in the relationships between Pearson residuals and the fitted counts and predictor variables (Figure 7).

**Table 8:** Model selection and model checking

	AIC		Cell counts			Correlation		Calibration		Error	
Model	BH	FH	y	ŷ	ŷ-y	r	R	*b*	*m*	AVE_error_	RMSE
Scale 1	19932.58	18661.30*	9000	9376	376	0.16	0.33	0.16	0.57	0.02	2.86
Scale 2	18273.88	17722.50*	9000	9438	438	0.16	0.35	0.29	0.54	0.03	3.71

Model selection and estimates of correlation, calibration and error used for the evaluation of the Scale 1 and Scale 2 hurdle models. * alongside the AIC values indicate the final models.

BH = Broad Habitat, FH = Fine Habitat; AIC = Akaike Information Criteria; y = observed, ŷ = predicted; r = Pearson correlation coefficient, R = Spearman rank correlation, *b *= intercept, *m *= slope; AVE_error _= average error, RMSE = root mean square error.

### Post-hoc investigations

To enhance our ability to interpret the model results in the context of the Cape Peninsula landscape, we conducted two post-hoc investigations to establish the spatial relationships between altitude and slope, and altitude and vegetation biomass. First, we used ArcView 3.3 to delineate the Cape Peninsula landscape into 100 m altitudinal belts increasing from sea level to 1100 m (regional maximum), and to calculate the mean (± 1SE) slope of each belt. Second, to analyse the relationship between altitude and vegetation biomass, we surveyed a section of the Cape Peninsula landscape. We assessed changes in biomass along three altitudinal transects running from sea level to 600 m above sea level. At 100 m intervals along each transect we visually determined the growth form and canopy cover of the dominant stratum within 10 × 5 m quadrates [*sensu *[76]]. These particular transects were selected because they were located in the only area of the Cape Peninsula that contained all of the following three attributes: (1) they included an extensive and traversable altitudinal range relative to other regions of the Cape Peninsula, (2) they covered an area of land stretching from sea level to mountain top that was undeveloped and dominated by natural habitat, and (3) they fell within a large enough area to allow three replicate surveys to be conducted while controlling for geology, hydrology and invasive alien vegetation.

Finally, using the Scale 1 model results, we calculated the absolute and cumulative areas of natural habitat remaining in the Cape Peninsula for each level of probability and each category of predicted abundance.

## Competing interests

The authors declare that they have no competing interests.

## Authors' contributions

TH and JO conceived of, designed and coordinated the study and drafted the manuscript. TH collected the data and performed the statistical analysis. Both authors read and approved the final manuscript.
